# Thrombolysis in Acute Ischemic Stroke: A Simulation Study to Improve Pre- and in-Hospital Delays in Community Hospitals

**DOI:** 10.1371/journal.pone.0079049

**Published:** 2013-11-18

**Authors:** Maarten M. H. Lahr, Durk-Jouke van der Zee, Patrick C. A. J. Vroomen, Gert-Jan Luijckx, Erik Buskens

**Affiliations:** 1 Department of Neurology, University Medical Center Groningen, University of Groningen, Groningen, The Netherlands; 2 Department of Operations, Faculty of Economics & Business, University of Groningen, Groningen, The Netherlands; 3 Health Technology Assessment, Department of Epidemiology, University Medical Center Groningen, University of Groningen, Groningen, The Netherlands; University of Muenster, Germany

## Abstract

**Background:**

Various studies demonstrate better patient outcome and higher thrombolysis rates achieved by centralized stroke care compared to decentralized care, i.e. community hospitals. It remains largely unclear how to improve thrombolysis rate in decentralized care. The aim of this simulation study was to assess the impact of previously identified success factors in a central model on thrombolysis rates and patient outcome when implemented for a decentral model.

**Methods:**

Based on a prospectively collected dataset of 1084 ischemic stroke patients, simulation was used to replicate current practice and estimate the effect of re-organizing decentralized stroke care to resemble a centralized model. Factors simulated included symptom onset call to help, emergency medical services transportation, and in-hospital diagnostic workup delays. Primary outcome was proportion of patients treated with thrombolysis; secondary endpoints were good functional outcome at 90 days, Onset-Treatment-Time (OTT), and OTT intervals, respectively.

**Results:**

Combining all factors might increase thrombolysis rate by 7.9%, of which 6.6% ascribed to pre-hospital and 1.3% to in-hospital factors. Good functional outcome increased by 11.4%, 8.7% ascribed to pre-hospital and 2.7% to in-hospital factors. The OTT decreased 17 minutes, 7 minutes ascribed to pre-hospital and 10 minutes to in-hospital factors. An increase was observed in the proportion thrombolyzed within 1.5 hours; increasing by 14.1%, of which 5.6% ascribed to pre-hospital and 8.5% to in-hospital factors.

**Conclusions:**

Simulation technique may target opportunities for improving thrombolysis rates in acute stroke. Pre-hospital factors proved to be the most promising for improving thrombolysis rates in an implementation study.

## Introduction

Treatment with tissue plasminogen activator (tPA) or thrombolysis is the most effective therapy for acute ischemic stroke patients within the first 4.5 hours following the onset of stroke symptoms [1], [2]. However, thrombolysis is substantially underused. Of all stroke patients, currently between 1–8% [3]–[5] are treated with tPA worldwide and around 11% (ranging from 4–26%) within the Netherlands [6], while 24–31% may be achieved in optimized settings [7], [8]. Reasons for this undertreatment are multi-factorial. Patients may not be familiar with the symptoms of a stroke or may not know how to act. Also, access to stroke care and expertise may vary by location [9], [10]. Other factors influencing the use of thrombolysis ensue from the organization of acute stroke care. Recently we demonstrated a 50% greater likelihood and up to 22% overall rate of treatment with thrombolysis achieved by a stroke center in a centralized organizational model versus nine community hospitals united in a decentralized organizational system of acute stroke care [11].

So far, it remains largely unclear how to improve thrombolysis delivery in decentralized stroke care; i.e. community hospitals. A method to study improvement of thrombolysis delivery is using Randomized Controlled Trials (RCTs). Although the benefits of RCTs have been clearly established in case of single interventions, their efficacy may be limited in case of complex patient pathways such as thrombolysis. For example, two recently published RCTs showed nonsignificant increases in thrombolysis rate following an intensive multicomponent intervention [12], [13]. Taken together, these studies focused on singular aspects of thrombolysis delivery (i.e. the in-hospital phase) and seem to yield very little gains- in terms of an increase in thrombolysis use- for the time and money invested. This warrants the question whether alternative research strategies are needed to study delivery of thrombolysis.

Various studies have shown how simulation models may be used as an efficient alternative or precursor to clinical trials, for example by predicting the prognosis after aortic heart valve replacement before implementation of the therapy [14], and studying the clinical benefit of reducing in-hospital delays to maximize the population benefit of thrombolysis in acute ischemic stroke [15]. As opposed to RCTs, simulation models allow for studying the effect of multiple factors along the entire stroke pathway at high pace and low costs.

The aim of this simulation study was to assess the impact of previously identified success factors in a central model on thrombolysis rates and patient outcome when implemented for a decentral model.

## Methods

### Study setting and patients

In the North of The Netherlands a centralized and a decentralized organizational model co-exist. The centralized model covers the catchment area of 4 hospitals, in which thrombolysis is only provided by University Medical Center Groningen (UMCG) acting as a designated stroke center. The decentralized model is adopted by 9 community hospitals all providing thrombolysis for their own catchment area. From February 1 to July 31 2010 all ischemic stroke patients admitted or referred to hospitals were registered. For both models identical protocols for tPA treatment (adjusted ECASS III [16]), identification and triage of suspected stroke patients, and 911 systems were used. Further details on population densities and access to healthcare services are provided in a previously published paper [11].

### Simulation model

A simulation model was developed using Plant Simulation software [17]. Factors in which the central model performed significantly better than the decentral model were incorporated in the simulation model: lapse between symptom onset to call for help, first responder; i.e. 911 or General Practitioner (GP), Emergency Medical Services (EMS) transport, high priority transport by EMS, and the time from hospital arrival to neurological- and neuroimaging (Computed Tomography, CT) examination. Data collected in a previously performed prospective study of 801 diagnosed ischemic stroke patients admitted in a decentralized model and 283 in a centralized model were used as input for the simulation model [11]. We aimed to simulate current practice and compare it with scenarios in which we implemented success factors of the central model in the decentral model. Next, the impact on thrombolysis rates and patient outcome were assessed for single factors and combinations thereof. Probability distributions derived from empirical data were used to model event rates, activity durations and diagnostic accuracy. Details on stroke pathway setup, simulation methodology, and model data are provided in Text S1. In the model, 10,000 patients progressed along the stroke pathway.

### Data collection

Data of ischemic stroke patients were collected for both the pre-hospital and in-hospital phase by ambulance personnel and experienced stroke neurologists. All data were entered directly into a web-based database to ensure high quality of data. Patients not transported by EMS were referred by the GP and arrived at the hospital by self transport in case no longer eligible for thrombolysis and the medical condition allowed such (i.e. stable or not).

### Outcome measures

The primary end-point was the proportion of patients treated with thrombolysis. Secondary end-points were proportion of patients with good functional outcome at 90 days (modified Rankin Scale 0–1), Onset-Treatment-Time (OTT), and shift of OTT to a shorter time window, because the benefit of thrombolysis is strongly time dependent, the sooner the better.

### Statistical analysis

Mann-Whitney *U* tests and Chi-square tests were performed for continuous and categorical variables. A p-value <0.05 was considered statistically significant. SPSS 20.0 for windows software package (Chicago, Il) was used.

### Informed consent

Informed consent was obtained from all subjects participating in the previous observational study and extended for current use [11]. Written consent was given by the patients for their information to be stored in the hospital database and used for research. The study was approved by the institutional review board of the University Medical Center Groningen.

## Results

### Observational study – performance for organizational models

In the observational study, the difference in thrombolysis rate between the decentralized and centralized model was 7.8% (14.1% vs. 21.9%, respectively). Table 1 describes the performance for each factor investigated in both models, and Table 2 shows the results of the nine scenarios selected for this study on primary and secondary outcome measures. Details on the distributions underlying activity durations and diagnostic accuracy of both the decentralized- and centralized organizational model are presented in Table S1 and Table S2, respectively. Key activities of the acute stroke pathway and treatment decision are presented in Figure S1 and Figure S2, respectively.

**Table 1:** Organizational model performance for the pre- and in-hospital acute stroke pathway.

	Centralized model	Decentralized model
N	283	801
**Pre-hospital phase**		
Symptom onset to call for help time, valid cases	152 (53.7)	249 (31.1)*
Median (min)	40.5	36.5
		
First responder		
General practitioner (%)	135 (47.7)	456 (56.9)†
911 (%)	84 (29.7)	184 (23.0)†
		
Transported by EMS (%)	213 (75.3)	462 (57.8)*
High priority transportation by EMS (%)	170 (79.8)	310 (67.1)*
		
**In-hospital phase**		
In-hospital diagnostic workup, median (min)		
Time to neurological examination	0.0	4.0*
Time to neuroimaging examination	8.0	22.0*

EMS indicates emergency medical services.

* P<0.01.

†P<0.05.

**Table 2:** Re-configuration decentralized model: results simulation experiments.

	tPA rate (95% CI)	OTT min (95% CI)	tPA 0–1.5 hr (95% CI)	tPA 1.5–3.0 hr (95% CI)	tPA 3.0–4.5 hr (95% CI)	mRS 0–1‡
**Scenario**						
0. Current practice	14.4% (13.7% –15.1%)	134 (63–235)	14.3% (12.6% –16.3%)	70.5% (68.1% –72.8%)	15.2% (13.4% –17.1%)	14.7%
**Pre-hospital phase**						
1. Symptom onset to call for help	16.1% (15.4% –16.8%)	128 (50–238)	21.0% (19.0% –23.0%)	64.7% (62.3% –67.0%)	14.4% (12.7% –16.2%)	17.5%
2. First responder	15.3% (14.6% –16.0%)	130 (63–235)	15.5% (13.8% –17.4%)	71.8% (69.5% –74.0%)	12.7% (11.1% –14.5%)	15.9%
3. EMS transport	17.2% (16.4% –17.9%)	134 (67–235)	13.2% (11.7% –14.9%)	73.0% (70.9% –75.1%)	13.8% (12.2% –15.5%)	17.5%
4. High priority transport by EMS	14.6% (13.9% –15.3%)	133 (64–240)	14.8% (13.1% –16.7%)	70.1% (67.7% –72.4%)	15.1% (13.4% –17.1%)	15.0%
5. Combining all pre-hospital scenarios	20.8% (20.0% –21.6%)	127 (44–241)	20.8% (19.1% –22.6%)	66.2% (64.2% –68.2%)	13.0% (11.6% –14.5%)	22.7%
**In-hospital phase**						
6. Neurological examination	14.9% (14.2% –15.6%)	130 (63–235)	17.1% (15.3% –19.1%)	69.9% (67.6% –72.2%)	13.0% (11.4% –14.8%)	15.7%
7. Neuroimaging examination	15.2% (14.5% –15.9%)	127 (56–232)	20.0% (18.1% –22.1%)	66.9% (64.5% –69.2%)	13.1% (11.5% –14.9%)	16.4%
8. Combing both in-hospital scenarios	15.4% (14.8% –16.2%)	124 (55–230)	22.8% (20.8% –25.0%)	65.5% (63.1% –67.9%)	11.7% (10.2% –13.4%)	17.2%
9. Combining all scenarios	22.3% (21.5% –23.1%)	117 (32–236)	28.4% (26.8% –30.3%)	61.2% (59.2% –63.2%)	10.4% (9.3% –11.8%)	26.1%

tPA indicates tissue plasminogen activator; CI, confidence interval; OTT, onset-treatment-time; mRS, modified rankin scale; EMS, emergency medical services.

‡Indicates the proportion of patients with good outcome (mRS 0–1) ascribed to treatment with thrombolysis. The number needed to treat to achieve one patient with mRS 0–1 at 90 days for OTT 0–90 = 4.5, OTT 91–180 = 9.0, OTT 181–270 = 14.1 [21].

### Primary outcome

Compared to current practice, improving all factors to the level of the central model might increase thrombolysis rate by 7.9%, of which 6.6% ascribed to pre-hospital factors, and 1.3% to in-hospital factors. Of all pre-hospital factors, improving lapse between symptom onset to call for help contributed 1.7% to the total effect, 911 calls 0.9%, EMS transport 2.8%, and high priority EMS transport 0.2%. The remaining 1.0% is caused by an interaction effect between symptom onset to call for help and EMS transport. Of all in-hospital factors, 0.5% can be attributed to neurological examination, and 0.8% to neuroimaging examination.

### Secondary outcomes

**Figure 1:**
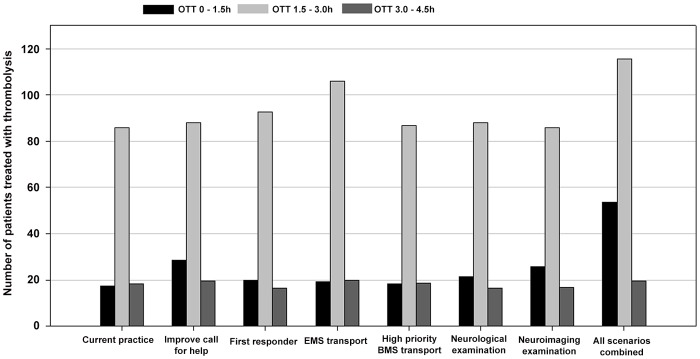
Number of patients treated with thrombolysis according to onset-treatment-time (OTT) intervals.

**Figure 2:**
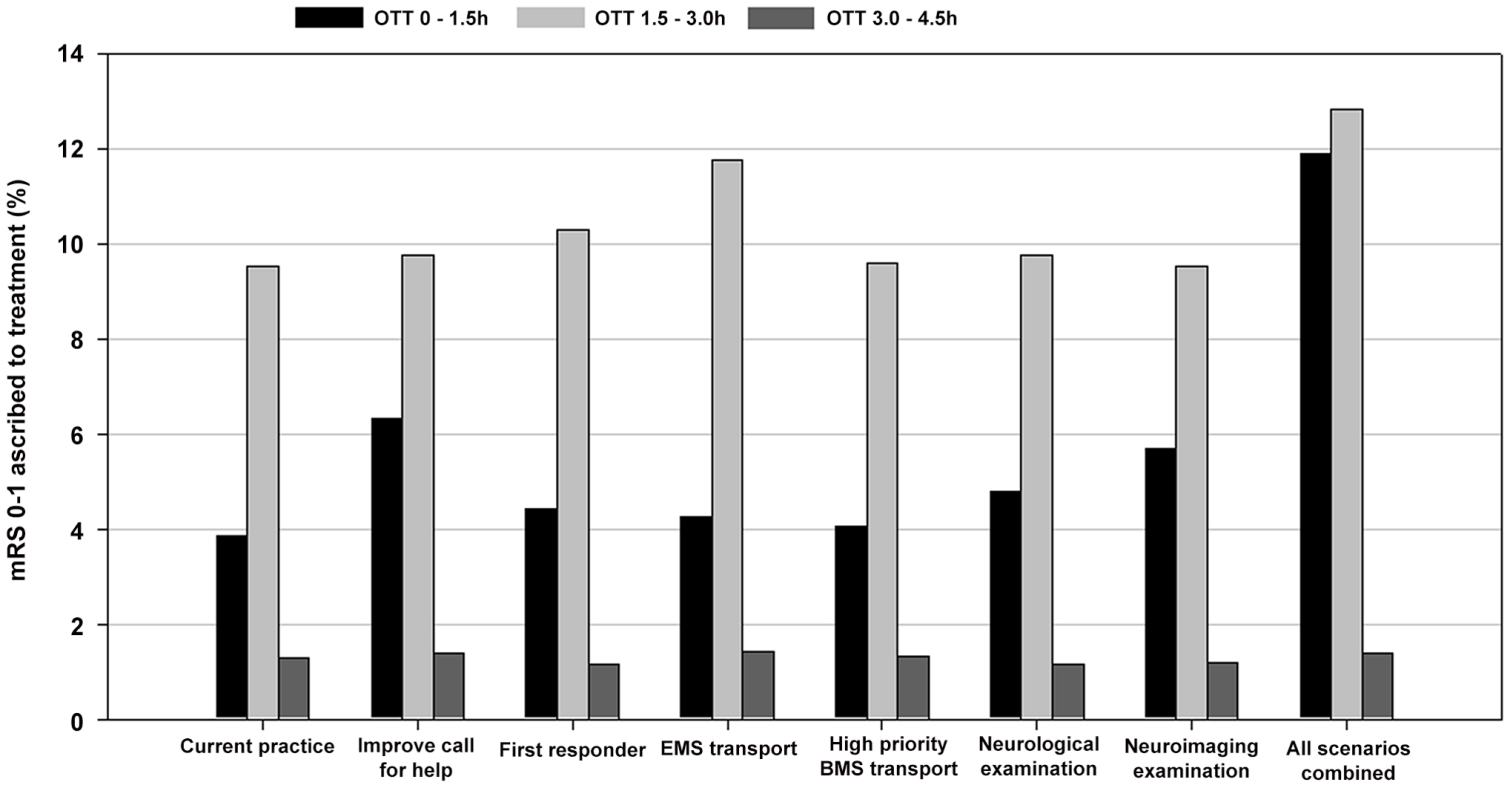
Patient outcome at 90 days for onset-treatment-time (OTT) intervals.

Improving all factors led to an 11.4% increase in patients with good functional outcome, of which 8.7% ascribed to pre-hospital factors and 2.7% to in-hospital factors. The OTT decreased 17 minutes; of which 7 minutes ascribed to pre-hospital factors and 10 minutes to the in-hospital factors. A shift was observed in the proportion of patients thrombolyzed within 1.5 hours; increasing by 14.1%, of which 5.6% ascribed to pre-hospital factors, and 8.5% to in-hospital factors. Treatment between 1.5–3.0 and 3.0–4.5 hours decreased by 9.3% and 4.8%, respectively; of which 4.3% and 2.2% attributed to pre-hospital factors and 5.0% and 2.6% to in-hospital factors, respectively. Figures 1 and 2 show the proportion of patients treated with thrombolysis according the OTT intervals and the proportion of patients with good functional outcome, respectively.

## Discussion

This simulation study quantifies the potential benefit of implementing success factors from a centralized organizational model in a decentralized model. Combining all factors raised thrombolysis rates by 7.9% thereby closing the gap observed between both models. Furthermore, our model demonstrates that the proportion of patients with a good functional outcome (mRS 0–1) may increase by 11.4% and those thrombolyzed within 1.5 hours by 14.1%.

This simulation study provides an example of an alternative research method to study delivery of thrombolysis in acute ischemic stroke. Contrary to traditional approaches that evaluate improvement strategies (i.e. RCTs), simulation models may be better suited to investigate multiple factors of stroke system organization simultaneously and allow for quicker answers at lower costs [15]. Instead of time-consuming and expensive trials such as double-blinded randomized trials, we advocate action research. This means implementing critical success factors determined from the simulation study in the existing acute stroke pathway and study the effects of interventions with baseline measurements. Simulation experiments obviously precede implementation to check whether comparable results can actually be achieved in clinical practice. By using the results obtained from simulation, implementation studies may be tailored towards improving factors impacting most on thrombolysis rates and patient outcome, while excluding others that do not.

Based on the identified potential for improving thrombolysis rates and patient outcomes pre-hospital factors seem primary targets for change. The importance of the pre-hospital phase in advancing the acute stroke care pathway is supported by a recent review article emphasizing that every link in the care pathway matters and should be studied for potential improvements [18]. In addition, we observed an interaction effect between improving symptom onset to call for help and EMS transport. This interaction effect may be explained by a volume effect – meaning more patients arriving at the hospital in time for thrombolysis, and by shortening the time to treatment for those already thrombolyzed. As treatment rates with thrombolysis are relatively high in our study compared to international standards, we would expect even larger effects when implemented in regions with low treatment rates.

Our study has limitations. Firstly, we were limited by the documentation of symptom onset times by ambulance and hospital personnel, i.e. possibly some of the ‘unknown’ onset times were actually known but never documented. However, the proportion of patients with unknown or estimated onset times was comparable to or even better than in previous studies [19], [20]. Secondly, the interaction between hospital size, i.e. stroke center and community hospitals, may be difficult to interpret because requirements of stroke centers are such that community hospitals are unlikely to meet them.

## Conclusions

This study demonstrates that simulation may be employed as research tool to target opportunities for improving the acute stroke care pathway and therefore thrombolysis rates. Pre-hospital factors proved to be the most promising targets for improving acute stroke care in an implementation study.

## Supporting Information

Figure S1 **Acute stroke pathway: key activities.**
(DOCX)

Figure S2 **Treatment decision: a patient's chance of being treated given the overall time delay.**
(DOCX)

Table S1 **Distributions specifying activity durations and diagnostic characteristics for the decentralized model.** Route 1, 2, and 3 indicate patients transported by emergency medical services, patients arriving by self transport, and those suffering a stroke in the hospital, respectively; GP, general practitioner; A1, A2, B indicate normative values for ambulance arrival within 15, 30, and >30 minutes from the 911 call until arrival at the location of the patients, respectively; CT, computated tomography; tPA, tissue plasminogen activator; EMS, emergency medical services. Neurological examination, neuroimaging, and laboratory examination are considered parallel activities.
(DOCX)

Table S2 **Distributions specifying activity durations and diagnostic characteristics for the centralized model.** Route 1, 2, and 3 indicate patients traversing the entire pathway, those patients suffering a stroke while being hospitalized, and patients arriving at the hospital by self referral, respectively; GP, general practitioner; A1, A2, B indicate normative values for ambulance arrival within 15, 30, and >30 minutes from the 911 call until arrival at the location of the patients, respectively; EMS, emergency medical services. Neurological examination, neuroimaging, and laboratory examination are considered parallel activities.
(DOCX)

Text S1 **Supplemental methods and results.**
(DOC)
