# Supplementary figures and images for: Thrombolysis in Acute Ischemic Stroke: A Simulation Study to Improve Pre- and in-Hospital Delays in Community Hospitals
Source: PLoS One. 2013 Nov 18;8(11):e79049. doi: 10.1371/journal.pone.0079049 (PMC3832502; doi:10.1371/journal.pone.0079049)

**Figure S1.** Acute stroke pathway: key activities.


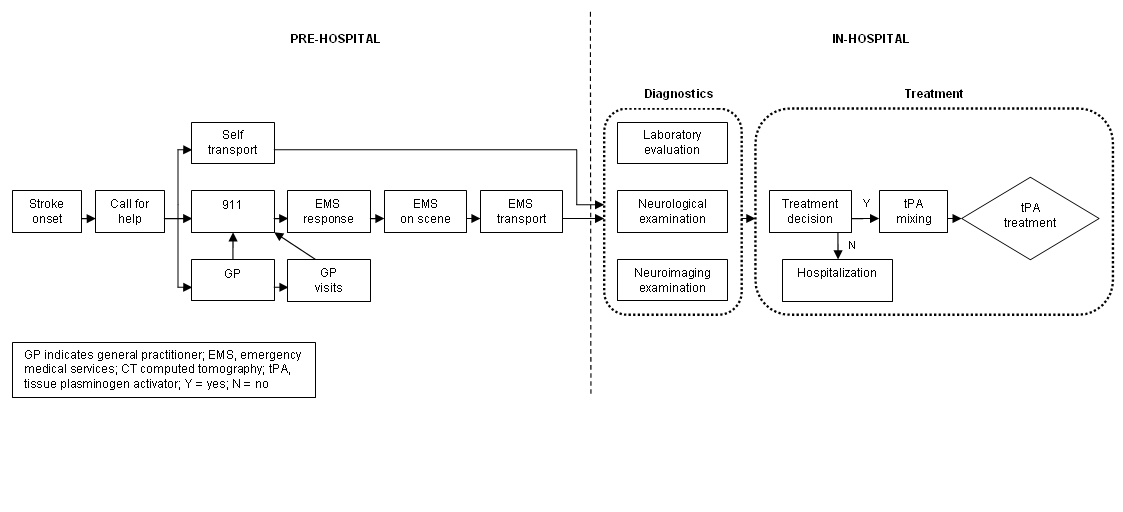

Supplement: Figure S1 — Acute stroke pathway: key activities. (DOCX) [file pone.0079049.s001.docx]

**Figure S2.** Treatment decision: a patient’s chance of being treated given the overall time delay.


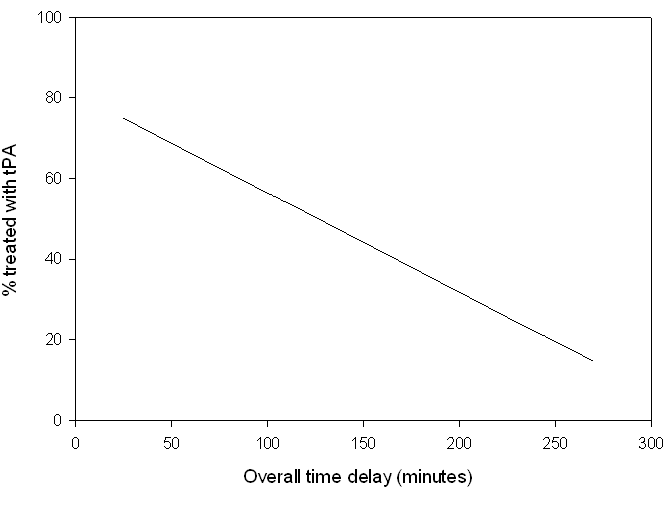

Supplement: Figure S2 — Treatment decision: a patient's chance of being treated given the overall time delay. (DOCX) [file pone.0079049.s002.docx]
