# Supplementary material for: Thrombolysis in Acute Ischemic Stroke: A Simulation Study to Improve Pre- and in-Hospital Delays in Community Hospitals
Source: PLoS One. 2013 Nov 18;8(11):e79049. doi: 10.1371/journal.pone.0079049 (PMC3832502; doi:10.1371/journal.pone.0079049)
Supplement: Table S1 — Distributions specifying activity durations and diagnostic characteristics for the decentralized model. Route 1, 2, and 3 indicate patients transported by emergency medical services, patients arriving by self transport, and those suffering a stroke in the hospital, respectively; GP, general practitioner; A1, A2, B indicate normative values for ambulance arrival within 15, 30, and >30 minutes from the 911 call until arrival at the location of the patients, respectively; CT, computated tomography; tPA, tissue plasminogen activator; EMS, emergency medical services. Neurological examination, neuroimaging, and laboratory examination are considered parallel activities. (DOCX) [file pone.0079049.s003.docx]

**Table S1.** Distributions specifying activity durations and diagnostic characteristics for the decentralized model.

| **Activity duration (minutes)** |  |  | | | | | |
| --- | --- | --- | --- | --- | --- | --- | --- |
| Model parameter | **Distribution: type** | **Parameters** | | | | | |
| Time from stroke onset to call for help  Route 1  Route 2  Route 3 | Continuous empirical | Left bound  0  5  10  15  30  45  60  120  180  240  480  0  60  120  180  240  480  0  5  10  480 | | Right bound  5  10  15  30  45  60  120  180  240  480  2880  60  120  180  240  480  2880  5  10  15  2880 | | | Frequency  39  21  15  35  33  14  40  16  10  9  230  9  17  3  4  2  286  1  1  1  15 |
| Delay first responder  911 call  GP consult by telephone  GP consult by visit | Uniform  Uniform  Triangle | Min (1.00), Max (2.00)  Min (2.00), Max (5.00)  Mode (40.00), Min (10.00), Max (30.00) | | | | | |
| Emergency Medical Services |  |  | | | | | |
| Response time  A1 | Continuous empirical | Left bound Right bound Frequency  0 5 53  5 10 145  10 15 85  15 20 23  20 25 2  25 30 1  30 35 0  35 40 2 | | | | | |
| A2 | Gamma | Alpha (3.36), Beta (4.22) | | | | | |
| B | Beta | Alpha 1 (0.69), Alpha 2 (0.53) , a (8.92), b (59.33) | | | | | |
| Time spent on scene  A1 | Continuous empirical | Left bound Right bound Frequency  0 5 3  5 10 45  10 15 106  15 20 87  20 25 39  25 30 13  30 35 8  35 40 3  40 45 4  45 60 2 | | | | | |
| A2 | Lognormal | Mean (15.24), St. dev. (7.67) | | | | | |
| B | Lognormal | Mean (15.12), St. dev. (8.14) | | | | | |
| Transport time  A1  A2  B | Beta  Gamma  Beta | Alpha1 (1.69), Alpha2 (4.16), a (39.66), b (0.62)  Alpha (5.53), Beta (2.51)  Alpha 1 (1.07), Alpha 2 (1.41), a (0.02), b (31.61) | | | | | |
|  |  |  |  | | |  | |
| Time to neurological consultation | Continuous empirical | Left bound  0  0  1  2  5  10  15  30 | Right bound  0  1  2  5  10  15  30  72 | | | Frequency  79  5  13  39  30  19  12  19 | |
| Time to neuroimaging (CT) examination | Continuous empirical | Left bound  0  5  10  15  20  25  30  35  40  45  50  55  85  90 | Right bound  5  10  15  20  25  30  35  40  45  50  55  85  90  135 | | | Frequency  9  23  36  29  32  29  21  11  11  3  2  7  3  2 | |
| Time to laboratory examination | Continuous empirical | Left bound  0  0  10  15  20  25  30  35  40  45  50 | Right bound  0  10  15  20  25  30  35  40  45  50  80 | | | Frequency  61  5  8  14  18  16  14  20  18  12  16 | |
| Treatment decision | Triangle | Mode (10), Min (5), Max (20) | | | | | |
| tPA mixing | Constant | 5 | | | | | |
|  |  |  | | | | | |
| **Diagnostics** |  |  | | | | | |
| Choice of route  1. EMS transport  2. In-hospital  3. Self-transport | Discrete empirical | Value  1  2  3 | | | Frequency  462  18  321 | | |
| Choice first responder  1. 911 call  2. GP consult by phone  3. GP consult by visit | Discrete empirical | Value  1  2  3 | | | Frequency  184  56  126 | | |
| EMS transport, level of urgency  911 call  1. A1  2. A2  3. B  GP consult by telephone  1. A1  2. A2  3. B  GP consult by visit  1. A1  2. A2  3. B | Discrete empirical | Value  1  2  3  1  2  3  1  2  3 | | | Frequency  92  7  1  60  39  1  47  42  11 | | |

**Table S1.** Distributions specifying activity durations and diagnostic characteristics for the decentralized model.

Route 1, 2, and 3 indicate patients transported by emergency medical services, patients arriving by self transport, and those suffering a stroke in the hospital, respectively; GP, general practitioner; A1, A2, B indicate normative values for ambulance arrival within 15, 30, and > 30 minutes from the 911 call until arrival at the location of the patients, respectively; CT, computated tomography; tPA, tissue plasminogen activator; EMS, emergency medical services. Neurological examination, neuroimaging, and laboratory examination are considered parallel activities.
