# Supplementary material for: Thrombolysis in Acute Ischemic Stroke: A Simulation Study to Improve Pre- and in-Hospital Delays in Community Hospitals
Source: PLoS One. 2013 Nov 18;8(11):e79049. doi: 10.1371/journal.pone.0079049 (PMC3832502; doi:10.1371/journal.pone.0079049)
Supplement: Table S2 — Distributions specifying activity durations and diagnostic characteristics for the centralized model. Route 1, 2, and 3 indicate patients traversing the entire pathway, those patients suffering a stroke while being hospitalized, and patients arriving at the hospital by self referral, respectively; GP, general practitioner; A1, A2, B indicate normative values for ambulance arrival within 15, 30, and >30 minutes from the 911 call until arrival at the location of the patients, respectively; EMS, emergency medical services. Neurological examination, neuroimaging, and laboratory examination are considered parallel activities. (DOCX) [file pone.0079049.s004.docx]

**Table S2.** Distributions specifying activity durations and diagnostic characteristics for the centralized model.

| **Activity duration (minutes)** | **Distribution** | **Parameters** | | | |
| --- | --- | --- | --- | --- | --- |
| Time from stroke onset to call for help  Route 1  Route 2  Route 3 | Continuous empirical | Left bound  0  5  10  15  30  45  60  120  180  240  480  0  120  240  480 | Right bound  5  10  15  30  45  60  120  180  240  480  2880  5  180  480  2880 | | Frequency  34  4  8  13  15  13  19  13  9  12  73  6  1  1  58 |
| Time to neurological consultation | Continuous empirical | Left bound  0  0  1  2  5 | Right bound  0  1  2  5  24 | | Frequency  93  4  7  6  12 |
| Time to neuroimaging examination | Continuous empirical | Left bound  2  6  11  16  21  31 | Right bound  5  10  15  20  30  56 | | Frequency  28  54  13  10  8  8 |
|  |  |  | | | |
| **Diagnostics** |  |  | | | |
| Choice of route  1. Entire stroke pathway  2. In-hospital  3. Self-referral | Discrete empirical | Value  1  2  3 | | Frequency  213  7  60 | |
| Choice first responder  1. 911 call  2. GP consult by phone  3. GP consult by visit | Discrete empirical | Value  1  2  3 | | Frequency  30  19  27 | |
| EMS transport, level of urgency  911 call  1. A1  2. A2  3. B  GP consult by telephone  1. A1  2. A2  3. B  GP consult by visit  1. A1  2. A2  3. B | Discrete empirical | Value  1  2  3  1  2  3  1  2  3 | | Frequency  95  3  2  88  10  2  60  33  7 | |

Route 1, 2, and 3 indicate patients traversing the entire pathway, those patients suffering a stroke while being hospitalized, and patients arriving at the hospital by self referral, respectively; GP, general practitioner; A1, A2, B indicate normative values for ambulance arrival within 15, 30, and > 30 minutes from the 911 call until arrival at the location of the patients, respectively; EMS, emergency medical services. Neurological examination, neuroimaging, and laboratory examination are considered parallel activities.
