# Supplementary material for: Thrombolysis in Acute Ischemic Stroke: A Simulation Study to Improve Pre- and in-Hospital Delays in Community Hospitals
Source: PLoS One. 2013 Nov 18;8(11):e79049. doi: 10.1371/journal.pone.0079049 (PMC3832502; doi:10.1371/journal.pone.0079049)
Supplement: Text S1 — Supplemental methods and results. (DOC) [file pone.0079049.s005.doc]

**Supporting information**

**Thrombolysis in acute ischemic stroke: A simulation study to improve**

**pre- and in-hospital delays in community hospitals**

Maarten M.H. Lahr MSc (Res), Durk-Jouke van der Zee PhD, Patrick C.A.J. Vroomen MD, PhD, Gert-Jan Luijckx MD, PhD, Erik Buskens, MD, PhD.

**Introduction**

The main text of the manuscript summarizes and discusses the most important findings of the study. This online supporting information details on simulation modeling methodology and model data.

**Supplemental Methods**

***Discrete event simulation***

The simulation model built conforms to the notion of discrete event simulation. Discrete Event Simulation (DES) entails the modeling of a system as it evolves over time by a representation in which variables change instantaneously at separate, i.e. discrete, points in time [1]. Because the acute stroke pathway incorporates a great deal of variation in activity durations and diagnostics, DES is considered an appropriate method for modeling such processes.

***Model validation***

Comparing simulation model outcomes with real system performance, thrombolysis rate in the model was 14.4% vs. 14.1% in the real system (P=0.87), and onset-treatment-time 127 vs. 134 minutes (P=0.12). Face validity was supported by two stroke neurologists (G-J.L., and P.C.A.J.V.) acting as domain experts.

***Distribution fitting***

Probability distributions for input parameters of the model were determined (fitted) using ExpertFit (Tables S1 and S2). Main steps involved:

- Importing real system data into ExpertFit.
- Fitting theoretical distributions by using the method of maximum likelihood [1].
- Seeking further evidence in case of a “no fit”, in an attempt to underpin the choice for a specific theoretical distribution. Evidence considered includes usage of the candidate distribution(s), commonalities between highest ranked distributions, and consultation of domain experts [2]. If such evidence is not found an empirical distribution was chosen.

***Set-up of experiments***

All experiments concern observations on 10,000 hypothetical patients. The number of patients is chosen such that the 95% confidence interval half width is below 1% of the mean treatment rate.

***Software***

Plant Simulation was used to model the stroke pathway [3]. Choice of probability distributions and their respective parameters is made using ExpertFit [4].

***Model data***

Model set-up corresponds to description of the acute stroke pathway (Figure S1). Patients are classified according to their choice of route towards the hospital (see Table S1 for the decentralized model and Table S2 for the centralized model). Next, traversing each entails sampling from distributions specifying activity durations. Note how activity durations may be moderated by diagnostic outcomes. Finally, cumulative delay for a patient is used as an input for the treatment decision. Here a larger delay implies a smaller chance of being treated.

**Supplementary Results**

***Treatment decision***

The efficacy of thrombolysis in acute ischemic stroke is greater the earlier it is administered, and the clinical benefit declines progressively over time [5]. For the simulation model the likelihood of treatment is approximated by a linear function, see Figure S2. We used a linear regression model (Y-axis intercept 82.1; slope -0.25) to approximate the chance of tPA treatment set against the overall time delay for all patients arriving < 4.5 hours from the onset of stroke symptoms (i.e. eligible for tPA treatment).

References

1. Law AM, Kelton WD. (2007) Simulation modeling and analysis. 4th ed. : McGraw-Hill.

2. Stahl JE, Furie KL, Gleason S, Gazelle GS. (2003) Stroke: Effect of implementing an evaluation and treatment protocol compliant with NINDS recommendations. Radiology 228: 659-668.

3. Plant simulation. siemens PLM 2012. Accessed July 12, 2013.

4. Law AM. (2011) ExpertFit version 8 user's guide. Tuscon, Arizona: Averill M. Law & Associates.

5. Lees KR, Bluhmki E, von Kummer R, Brott TG, Toni D, et al. (2010) Time to treatment with intravenous alteplase and outcome in stroke: An updated pooled analysis of ECASS, ATLANTIS, NINDS, and EPITHET trials. Lancet 375: 1695-1703.
